# Novel strategy using live non-pathogenic *Leishmania* expressing selected parasite antigens as a candidate vaccine for leishmaniasis

**Published:** 2011-01-10

**Authors:** Sima Rafati, Amir Mizbani, Tahere Taheri, Farnaz Zahedifard, Yasaman Taslimi, Barbara Papadopoulou

**Affiliations:** 1 Molecular Immunology and Vaccine Research Laboratory, Pasteur Institute of Iran, Tehran, Iran; 2 Research Centre in Infectious Diseases, CHUL Research Centre and Department of Medical Biology, Faculty of Medicine, Laval University, Quebec G1V 4G2, Canada

## 

Parasites of the genus *Leishmania* are intracellular protozoa which are transmitted to their mammalian host by the bite of infected sand flies and cause a group of diseases known as Leishmaniasis. Despite attempting different vaccination strategies, no human vaccine is yet available against this disease. There is increasing evidence that presence of a small number of live parasites is necessary to maintain durable immunity, and the only way to meet this requirement is by using attenuated live vaccines [1]. The main obstacle about attenuated live strains is the risk of reversion of the organism to its virulent state. Another approach to reach this strategy is to use non-pathogenic *Leishmania* such as *L. tarentolae*. This parasite is lizard parasite and has never been found associated with any leishmaniasis in humans and is considered as non-pathogenic to humans. Previous studies have shown that *L. tarentolae* can be used as a live vaccine against *L. donovani* and elicit a protective Th1 immune response [2]. Recently, by comparative genomic analysis and expression profiles of well-characterized virulence factors such as GP63, CPB, LPG3, Amastin and A2 between pathogenic *Leishmania* species (e.g. *L. major*, *L. infantum* and *L. braziliensis*) and non pathogenic *L. tarentolae* revealed that only A2 is absent at the level of DNA [3]. A recombinant *L. tarentolae* expressing the A2 protein was generated and its potential as a live vaccine against *L. infantum* infection in BALB/c mice was examined [4]. The A2 expressing recombinant parasites showed higher macrophage infectivity in comparison to *L. tarentolae* used as a control. Immunization (i.v. and i.p.) of BALB/c mice with recombinant *L. tarentolae* A2 elicited a strong protective immunity against virulent *L. infantum* challenge, manifested by a dramatic decrease in parasite burdens in the liver and the spleen of immunized mice. IFN-g production upon antigen stimulation indicated that protection is associated with a Th1 cell-mediated immunity accompanied by reduced levels of IL-5 production (the Th2 type response). Interestingly, although IFN-g production is also induced in groups of mice immunized with wild type *L. tarentolae*, cytokine levels are increased in the group immunized with the recombinant *L. tarentolae* A2 and especially when the vaccine regimen is administered via the i.p. route [4]. In continuation of these promising results, we are expanded this idea against *L. major* infection as a novel vaccine regimen by including two immunogenic parasite proteins (cysteine proteinases A and B, CPA/CPB).
